# The Current Collapse in AlGaN/GaN High-Electron Mobility Transistors Can Originate from the Energy Relaxation of Channel Electrons?

**DOI:** 10.1371/journal.pone.0128438

**Published:** 2015-06-03

**Authors:** Ling-Feng Mao, Huan-Sheng Ning, Jin-Yan Wang

**Affiliations:** 1 School of Computer & Communication Engineering, University of Science & Technology Beijing, 30 Xueyuan Road, Haidian District, Beijing 100083, P. R. China; 2 Institute of Microelectronics, Peking University, 100871, Beijing, P. R. China; Gazi University, TURKEY

## Abstract

Influence of the energy relaxation of the channel electrons on the performance of AlGaN/GaN high-electron mobility transistors (HEMTs) has been investigated using self-consistent solution to the coupled Schrödinger equation and Poisson equation. The first quantized energy level in the inversion layer rises and the average channel electron density decreases when the channel electric field increases from 20 kV/cm to 120 kV/cm. This research also demonstrates that the energy relaxation of the channel electrons can lead to current collapse and suggests that the energy relaxation should be considered in modeling the performance of AlGaN/GaN HEMTs such as, the gate leakage current, threshold voltage, source-drain current, capacitance-voltage curve, etc.

## Introduction

The AlGaN/GaN high-electron mobility transistor as a promising technology has recently emerged in the application of switch and radio frequency electronics. The conduction loss increasing with the current collapse phenomenon in GaN HEMTs is one of the application issues [1]. Note that the current collapse phenomenon in nitride based HEMTs is inevitable and serious [2–7]. The concept of “virtual gate” used to describe the phenomenon of electrons trapped by the traps at the corner of the gate can explain the current handling of the channel and the phenomenon of “knee walkout” [2]. Using bilayer gate dielectric in GaN HEMTs, the current collapse phenomenon can be suppressed [6]. Multi field-plate structures are also used for alleviating such a phenomenon and strengthening the voltage handling capacity of GaN HEMTs, but the mechanism to suppress the current collapse phenomenon in GaN HEMTs is needed to be clarified especially for devices working at high voltage [7]. Developing the physical picture to explain the current collapse phenomena in GaN HEMTs is helpful to understanding how it limits the reliability of GaN HEMTs. Thus any effort for trying to understanding current collapse phenomena in GaN HEMTs deserves further study, and we try to propose a possible new physical origin for such a phenomenon in this paper with the help of self-consistent solution to the coupled Poisson equation and Schrödinger equation including the quantum coupling effects and the energy relaxation of channel electrons.

## Theory

The electrons in solid can obtain kinetic energy from the applied electric field. This means that the electron temperature in a HEMT channel can rise much higher than the lattice temperature. Using the concept of the energy relaxation time, the electron temperature in a HEMT channel, can be described by the following equation [8]

$$T_{e}=T_{L}+\frac{2}{3}\frac{q\tau_{e}\mu_{e}}{k_{B}}E_{ch}^{2}$$ (1)

where *T*_e_ the electron temperature, *T*_L_ the lattice temperature, *k*_B_ Boltzmann constant, *τ*_e_ the energy relaxation time of electrons, *E*_ch_ is the applied electric field along the channel, *q* the electron charge, and *μ*_e_ the electron mobility.

Considering quantum coupling effects, the Schrödinger equations in the region of GaN and AlGaN of GaN HEMTs according to the method described in Ref. [9–10] can be written as

$$\left[-\frac{\hbar^{2}}{2m_{GaN}^{*}}\frac{\partial^{2}}{\partial z^{2}}+\phi \left(z\right)\right]\psi \left(z\right)=E_{z}^{GaN}\psi \left(z\right)$$ (2)

$$\left[-\frac{\hbar^{2}}{2m_{AlGaN}^{*}}\frac{\partial^{2}}{\partial z^{2}}+\left(\phi \left(z\right)-k_{B}\left(T_{L}+\frac{2q\tau_{e}\mu_{e}E_{ch}^{2}}{k_{B}}\right)\left(1-\frac{m_{GaN}^{*}}{m_{AlGaN}^{*}}\right)\right)\right]\psi \left(z\right)=E_{z}^{GaN}\psi \left(z\right)$$ (3)

where *z* direction is perpendicular to the AlGaN/GaN the interface, Ψ(*z*) is the wave function along *z* direction, *ϕ*(*z*) is the potential barrier along *z* direction, $E_{z}^{GaN}$ is the energy of the electron along *z* direction, $k_{B}\left(T_{L}+\frac{2q\tau_{e}\mu_{e}E_{ch}^{2}}{k_{B}}\right)$ is the transverse energy of the channel electron in the plane parallel to the AlGaN/GaN interface, *ћ* is the reduced Planck’s constant, $m_{GaN}^{*}$ and $m_{AlGaN}^{*}$ are the effective electron masses of GaN and AlGaN, respectively.

In a HEMT, the 1D (one dimensional) Poisson equation along *z* direction can be written as

$$\frac{\partial }{\partial z}\left[\varepsilon \left(z\right)\frac{\partial \varphi }{\partial z}\right]=-q\left[N_{D}^{+}\left(z\right)-N_{A}^{\_}\left(z\right)+p\left(z\right)-n\left(z\right)\right]$$ (4)

where *φ*(z) is the electrostatic potential, *ε*(z) is spatially varying dielectric constant, $N_{D}^{+}\left(z\right)$ and $N_{A}^{\_}\left(z\right)$ are the ionized donor and acceptor concentrations respectively, and *n*(z) and *p*(z) are the electron and hole densities, respectively. In this paper, the Poisson equation (Eq 4) and Schrödinger equations (Eqs 2 and 3) are self-consistently solved with the finite-difference method using a grid size of 0.1 Å [11]. We use the iteration to self-consistently solve the coupled Schrödinger and Poisson equations with the convergence criteria for the potential as 1×10^-5^ eV in this paper. The coupled Schrödinger and Poisson equations have been self-consistently solved over the region from the metal/AlGaN interface to the position in the GaN substrate that is 160 Å away from the AlGaN/GaN interface. Boltzmann statistics is adopted for the region outside of this coupled Schrödinger and Poisson equations’ region.

## Results and Discussion

The relative dielectric constant of 9.5 for GaN [12] and of 9.2 for Al_0.3_Ga_0.7_N [13], the band gap of 3.4 eV for GaN [14] and of 4.24 eV forAl_0.3_Ga_0.7_N [15], and the effective electron mass of 0.2m_0_ for GaN [16] have been used in the calculations. Using the assumption of a linear increase of the effective mass parameter in AlGaN alloys with increasing Al-content [17] and 0.48m_0_ of AlN [18], thus the effective electron mass of 0.28m_0_ for Al_0.3_Ga_0.7_N is used in this paper. The value of spontaneous electron density is the magnitude of 10^-6^C/cm^2^ [19], thus the polarization electron concentration of 2×10^-6^C/cm^2^ for GaN and of 4.69×10^-6^C/cm^2^ for Al_0.3_Ga_0.7_N have been used in the calculations. Note that the valence band offset between GaN and AlN is 0.70±0.24 eV [20] and the conduction band offset equals to the band gap difference between GaN and AlN minus the valence band offset. Thus the authors assumed a value for the conduction band offset between Al_0.3_Ga_0.7_N and GaN as calculated from interpolations the values from 0 to that between GaN and AlN, and the conduction band offset of 0.67 eV between Al_0.3_Ga_0.7_N and GaN is used in the calculations. 300 nm thick GaN, 4 nm thick Al_0.3_Ga_0.7_N and the channel length of 3 μm have been used in all calculations.

Both the mobility and the energy relaxation time are dependent on the electric field along the channel [21]. The electron temperature can be determined via Eq 1 when both the mobility and the energy relaxation time are obtained. Using the mobility and the energy relaxation time reported in the Table III in Ref. [21], the obtained electron temperature and the channel electric field satisfy an exponential relation ($T_{e}=T_{e0}+A_{1}\text{exp}\left(\frac{E_{ch}}{t_{1}}\right)$, *T*_e0_ = 743.29646, *A*_1_ = 45.72958, *t*_1_ = 20.31491, the channel electric field is in the unit of kV/cm, here *T*_e0_ denotes the electron temperature when channel electric field is zero, cm is the centimeter, kV is the kilovolt, and s is the second). The Adj. R-Square is 0.99997 in the above fitting. In the following, we use this fitted exponential relation to delineate the relation between the electron temperature and the channel electric field. Note that the effective electron masses are a function of channel electric field and such effective electron masses represent these masses in the plane parallel to the GaN/AlGaN interface, and the effective electron masses perpendicular to the GaN/AlGaN interface are needed in the self-consistently solution to the coupled Schrödinger equation and Poisson equation. Thus the constant effective electron masses perpendicular to the GaN/AlGaN interface for both GaN and AlGaN have been used in the calculations.

**Fig 1:**
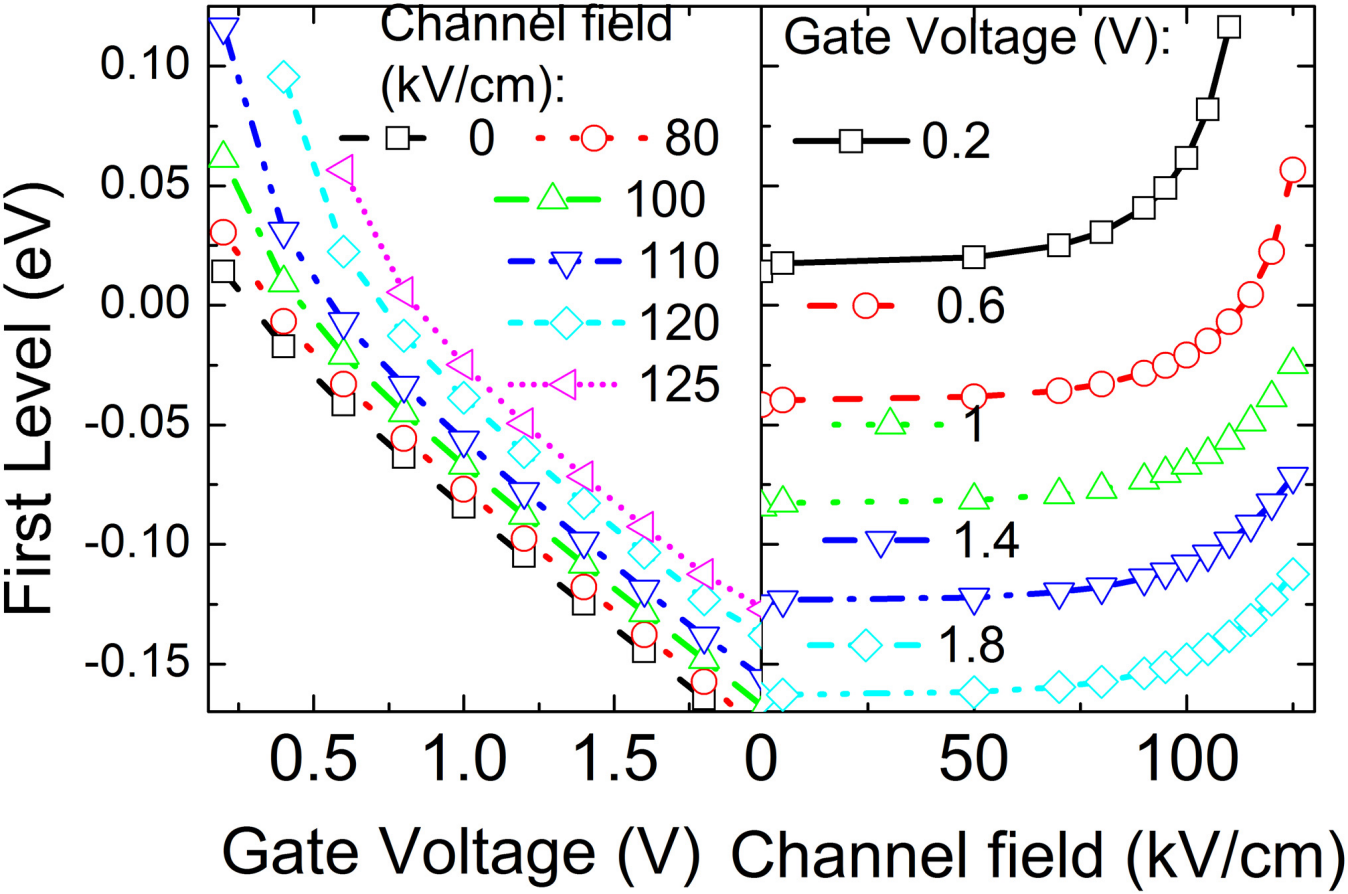
The first quantized level after considering the energy relaxation of the channel electrons as a function of the gate voltage and the channel electric field.

Fig 1 depicts how the first quantized energy level in the inversion layer after considering the energy relaxation of the channel electron changes with the electric field along the channel (gate voltage) at a given gate voltage (electric field along the channel). It can be clearly seen in this figure that the first quantized level drops when the gate voltage increases, whereas it increases when the channel electric field increasing. Note that the electron density in the GaN layer is determined according to Fermi statistics $n\left(z\right)=\frac{m_{GaN}^{*}k_{B}T}{\pi \hbar^{2}}\sum_{i}{\left|\psi_{i}\left(z\right)\right|}^{2}\text{ln}\left[1+e^{\left(E_{F}-E_{i}\right)/k_{B}T}\right]$. Here *E*_F_ is the Fermi level, *E*_*i*_ is the energy of the *i* ^*th*^ quantized level, *Ψ*_*i*_(*z*) is the electronic wave function along *z* direction that corresponds to the *i* ^*th*^ quantized level in the channel, *π* is the circumference ratio, and *T* is the device temperature. Thus a change in the quantized level will lead to a change in the inversion electron distribution. The reason why the energy relaxation of channel electrons has a large impact on quantized energy level in the inversion layer is because the energy relaxation of the channel electrons leads to a change in the Schrödinger equation in the AlGaN layer (see Eq 3), and the boundary conditions have been changed too when we self-consistently solve the coupled Schrödinger and Poisson equations. In other words, the barrier height at the GaN/AlGaN interface seen by the channel electrons will be reduced when the channel electrons get higher energy via the energy relaxation process. Thus the reduction in the barrier height at the GaN/AlGaN interface leads to a change in the quantized level and redistribute the channel electrons.

**Fig 2:**
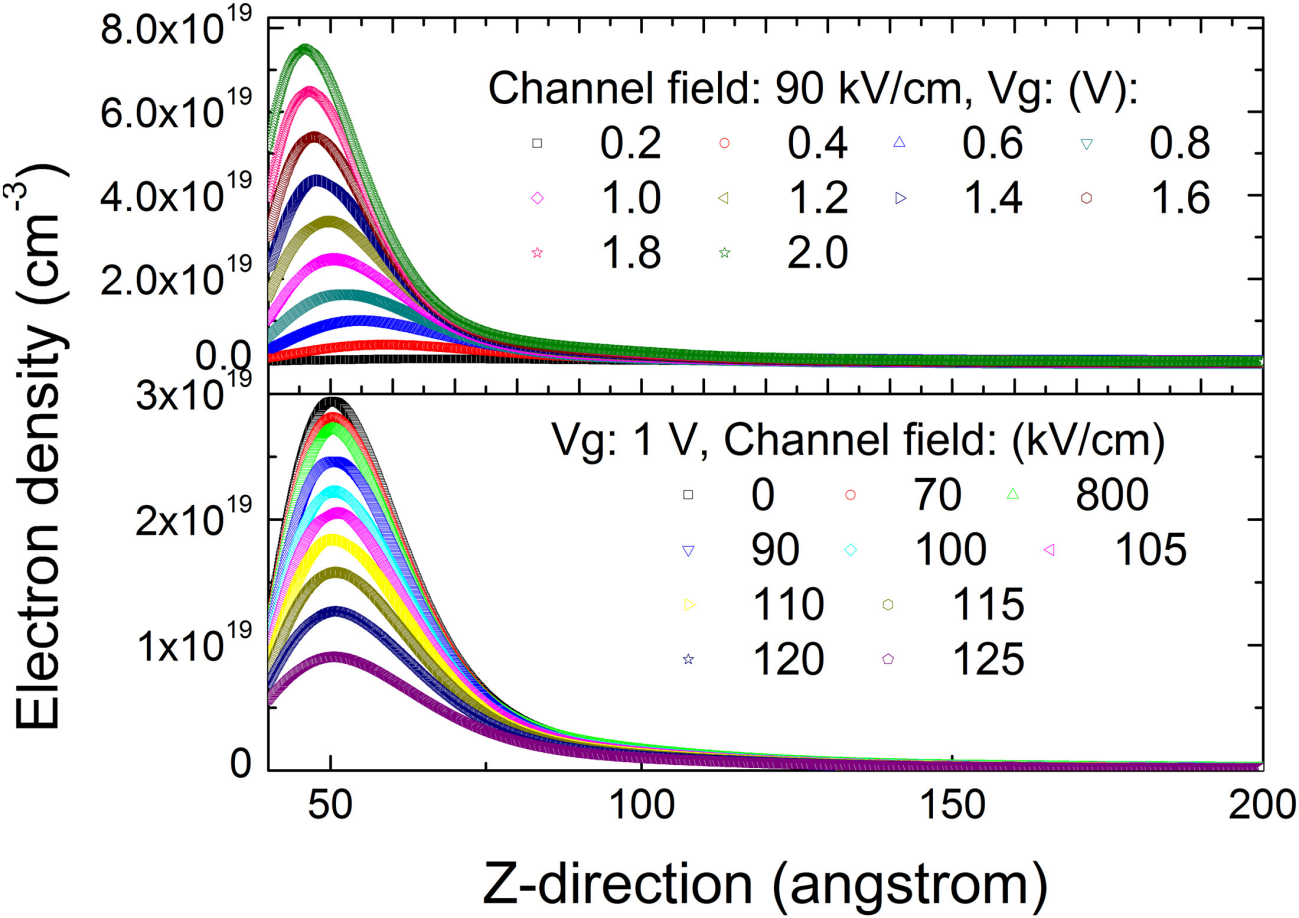
The distribution of the channel electron after considering the energy relaxation of the channel electrons for different gate voltage and channel electric field.

Fig 2 shows how the distribution of the channel electrons after considering the energy relaxation of the channel electron changes with the electric field along the channel (gate voltage) at a given gate voltage (electric field along the channel). This figure clearly illustrates that the peak of the channel electron density increases when the gate voltage increases, and the peak shift towards the AlGaN/GaN interface. It also shows that the peak of the channel electron density increases when the channel electric field increases, and the peak also shift toward the AlGaN/GaN interface. Both shifts are caused by the wave function of the quantized channel electrons. The reason why the energy relaxation of channel electrons has a large impact on the distribution of electron density in the inversion layer is because the energy relaxation of the channel electrons affects the quantization more. In other words, the reduction in the barrier height at the GaN/AlGaN interface caused by the energy relaxation of channel electrons cause a change in the quantized states, which means that both the quantized level and electronic wave-function change. Thus they have a large impact on the channel electron distribution along the direction perpendicular to GaN/AlGaN interface.

**Fig 3:**
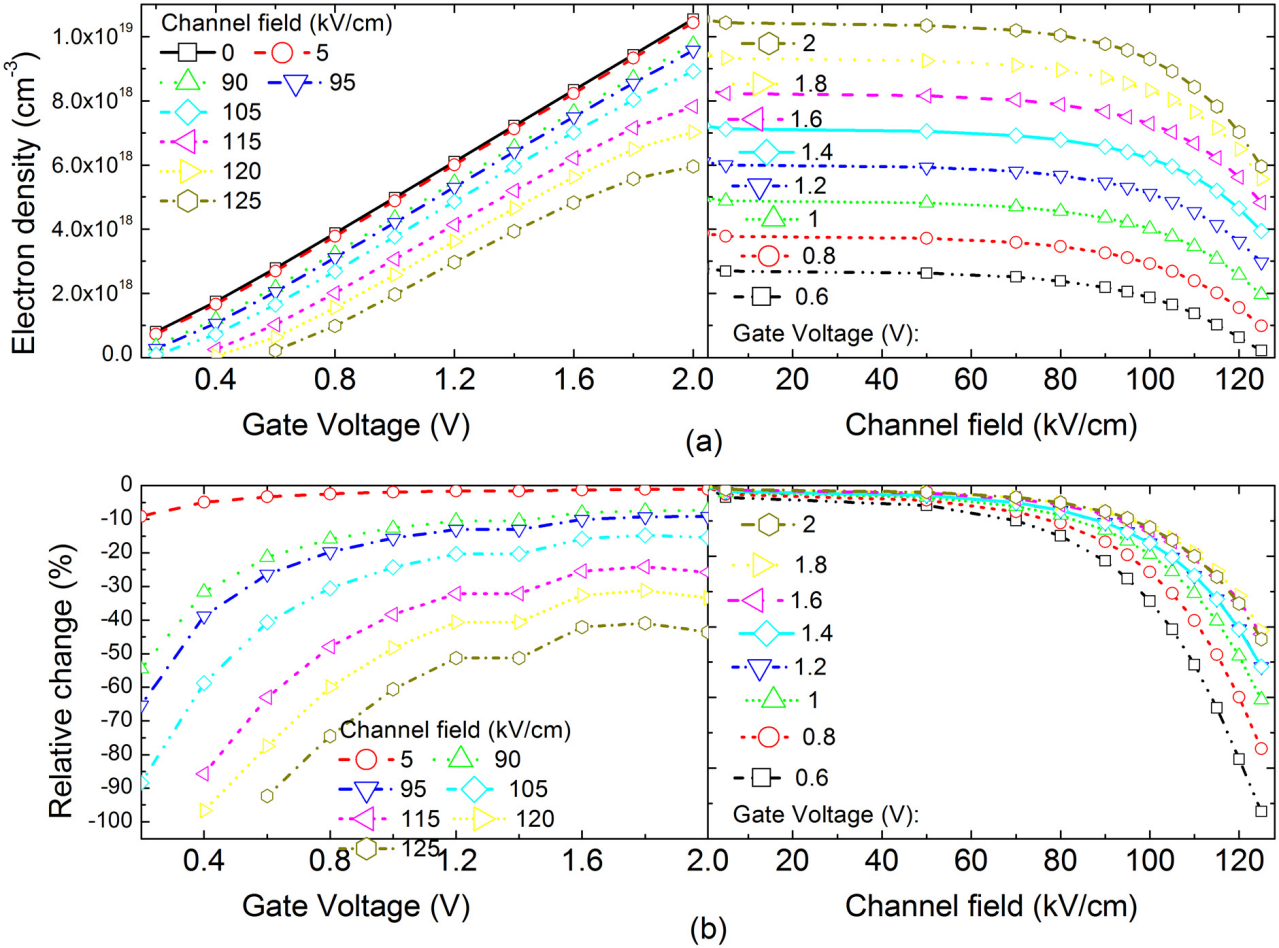
(a) The average electron density in the channel after considering the energy relaxation of the channel electrons as a function of the gate voltage and the channel electric field. (b) The relative increase in the average electron density in the channel considering the energy relaxation compared to those neglecting the energy relaxation.

Fig 3a compares the average channel electron density after considering the energy relaxation of the channel electrons under different gate voltage and channel electric field. This figure shows that the average electron density in the channel increases with the gate voltage, whereas the average electron density in the channel decreases with the channel electric field. That the increasing in the channel electric field reduces the average channel electron density implies that the increased drain voltage might decrease the saturation source-drain current. This leads to the current collapse phenomenon. In the following text, we will analyze the effects of the energy relaxation of the channel electrons on the source-drain current. Fig 3b compares the relative increase of the average channel electron density considering the energy relaxation with those neglecting the energy relaxation under the same conditions. Fig 3b clearly shows that the energy relaxation leads to the average channel electron density decreasing when the channel electric field increases. It implies that the energy relaxation may decrease the channel electron density and result in the current collapse phenomenon. In other words, the channel electric field have a large impact on the energy relaxation of channel electrons, thus the barrier height at the GaN/AlGaN interface seen by the channel electrons will be affected by changing the channel electric field. Thus its effects on the channel electron density can be determined via self-consistently solution to the coupled Schrödinger equation and Poisson equation.

Note that under constant mobility assumption, the source-drain current *I*_D_ can be written as [8]

$$\begin{array}{l} I_{D}=\frac{Z\mu }{L}\int_{0}^{L}Q_{n}\left(x\right)v\left(x\right)dx\\ =\frac{Z\mu }{L}C_{o}\left\{\left(V_{G}-V_{T}\right)V_{D}-\frac{V_{D}^{2}}{2}\right\} \end{array}$$ (5)

where *V*_T_ is the threshold voltage, *V*_g_ is the gate voltage, *V*_D_ is the drain voltage, *v* is the velocity of channel electron, *Q*_n_ is the channel electron density along the channel, $C_{o}=\frac{\varepsilon_{AlGaN}}{z_{0}+\Delta z}$, *z*_0_ is the thickness of AlGaN, Δ*z* is the channel thickness of the two-dimensional electron gas, *ε*_*GaN*_ and *ε*_*AlGaN*_ are the permittivity of GaN and AlGaN, respectively. For simplicity, we assume that the channel electric field along the channel is uniform and the energy relaxation of the channel electrons impacts on the inversion electron density along the channel has little change ($\frac{Q_{n}\left(y\right)}{Q_{n\_RE}\left(y\right)}\approx \frac{Q_{n}\left(y=0_{+}\right)}{Q_{n\_QC}\left(y=0_{+}\right)}=r\left(V_{g},V_{D}\right)$, where *y* is the direction along the channel, *r* is the ratio, *Q*_n_(*y*) and *Q*_*n_RE*_(*y*) are the inversion electron density without and with considering the energy relaxation of the channel electrons, respectively. Such a ratio can be directly obtained from the calculated channel electron density with considering the energy relaxation to that without at a give channel electric field (gate voltage) under different gate voltages (channel electric fields).

**Fig 4:**
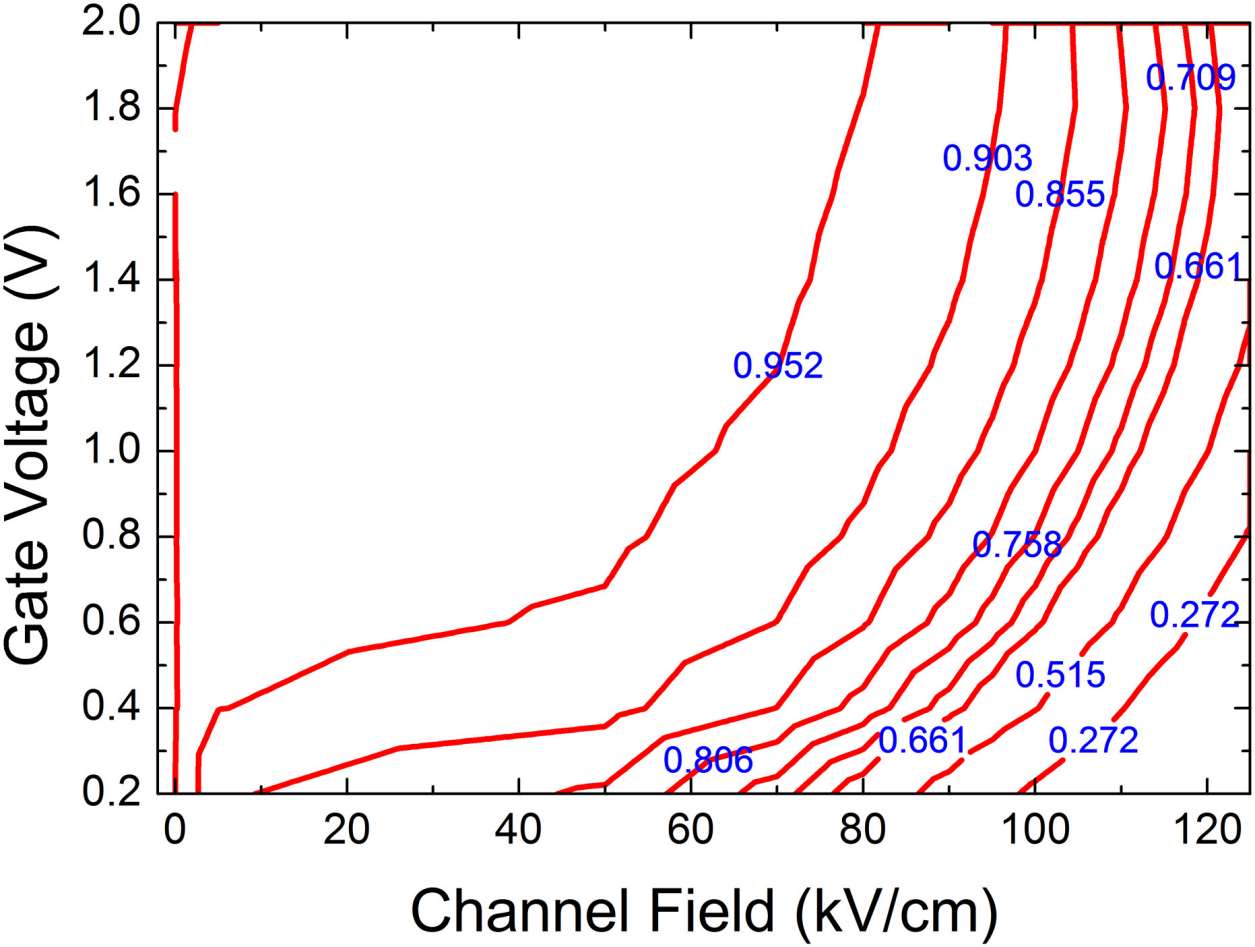
The contour of the ratio (*r*(*V*_*g*_,*V*_*D*_)) of the inversion electron density with considering the energy relaxation of the channel electrons to that without considering the energy relaxation of the channel electrons.

Fig 4 depicts the contour of the ratio of the inversion electron density with considering the energy relaxation of the channel electrons to that without considering the energy relaxation of the channel electrons. It is clearly seen in this figure that a higher channel electric field will cause a larger relative decrease in the inversion electron density. Thus the source-drain current with considering the energy relaxation of channel electrons *I*_*D_RE*_ can be simply discussed using the following expression,

$$\begin{array}{l} I_{D\_RE}=\frac{Z\mu }{L}\int_{0}^{L}r\left(V_{g},V_{D}\right)Q_{n}\left(x\right)v\left(x\right)dx\\ =r\left(V_{g},V_{D}\right)\frac{Z\mu }{L}C_{o}\left\{\left(V_{G}-V_{T}\right)V_{D}-\frac{V_{D}^{2}}{2}\right\} \end{array}$$ (6)

Using the electron mobility extracted from experiments at different channel electric field in the Table III in Ref. [21] and the threshold voltage determined by self-consistent solution to the coupled the Poisson (Eq 4) and Schrödinger equations (Eqs 2 and 3), the source-drain saturation current determined by Eq 6 should decrease with the drain voltage increasing because *r*(*V*_g_,*V*_*D*_) decrease when the channel electric field increases. In other words, when source-drain current saturates at a given channel electric field with a constant voltage applied to the gate, the decrease in the channel electron density caused by the increase in the channel electric field (which is described by the term *r*(*V*_g_,*V*_*D*_) introduced in Eq 6 and Fig 4 clearly show that a larger channel electric field a smaller *r*(*V*_g_,*V*_*D*_)) can lead to a decrease in the saturation current that is described as the current collapse phenomenon.

Note non-equilibrium between electron and lattice results that the electron energy (electron temperature) is much higher than lattice energy (lattice temperature), and it can be calculated via the electro-thermal analysis. The measured electron temperature can be higher than 2500 K and increases with the channel electric field increases in AlGaN/GaN HEMTs [22]. Under this operation conditions, the energy relaxation of the channel electrons in nitride based HEMT must be considered when the transverse energy of the channel electron in the plane parallel to the AlGaN/GaN interface ($k_{B}\left(T_{L}+\frac{2q\tau_{e}\mu_{e}E_{ch}^{2}}{k_{B}}\right)$) is enough large. Using an electro-thermal particle based device simulator, Ref. [23] shows that self-heating leads to the current degradation of about 11%. Our physics based mechanism for the current collapse phenomenon reported in the paper is then consistent with both experiment [22] and computer simulation [23].

## Conclusion

To summarize, the source-drain current degradation in AlGaN/GaN HEMTs can origin from the energy relaxation of the channel electrons. The average channel electron density is found to decrease when the channel electric field increases due to the energy relaxation of channel electrons. This relation between the average channel electron density and the channel electric field can explain the current collapse phenomenon in AlGaN/GaN HEMTs. It implies that further physical understanding of the energy dissipation and transport in AlGaN/GaN HEMTs is of great importance for device design and applications.
